# Quality of Life and Symptom Burden among Chronic Kidney Disease of Uncertain Etiology (CKDu) Patients in Girandurukotte, Sri Lanka

**DOI:** 10.3390/ijerph17114041

**Published:** 2020-06-05

**Authors:** Hansani Madushika Abeywickrama, Swarna Wimalasiri, Yu Koyama, Mieko Uchiyama, Utako Shimizu, Nahoko Kakihara, Rohana Chandrajith, Nishantha Nanayakkara

**Affiliations:** 1 Graduate School of Health Sciences, School of Health Sciences, Faculty of Medicine, Niigata University, 2-746, Asahimachi, Niigata 951-8518, Japan; uchiyama@clg.niigata-u.ac.jp (M.U.); shirakaba@clg.niigata-u.ac.jp (U.S.); kakihara@clg.niigata-u.ac.jp (N.K.); 2 Department of Food Science and Technology, Faculty of Agriculture, University of Peradeniya, Kandy 20400, Sri Lanka; swarnaw@pdn.ac.lk; 3 Department of Geology, Faculty of Science, University of Peradeniya, Kandy 20400, Sri Lanka; rohanac@pdn.ac.lk; 4 Transplant and Dialysis Unit, Teaching Hospital, Kandy 20000, Sri Lanka; nishantha4313@gmail.com

**Keywords:** health-related quality of life, symptom burden, chronic kidney disease of uncertain etiology, Sri Lanka

## Abstract

Symptom burden and health-related quality of life (HRQOL) are important predictors of how a disease affects patients’ lives, especially for endemic health problems such as chronic kidney disease of uncertain etiology (CKDu). Our study describes symptom burden, HRQOL, and associated demographic and clinical variables in CKDu patients in the Girandurukotte area, Sri Lanka. A cross-sectional study included 120 CKDu patients attending the renal clinic in the endemic area. The instruments applied were the Kidney Disease Quality of Life—Short Form (KDQOL-SF^TM^) version 1.3 and CKD Symptom Index—Sri Lanka. Socio-demographic, disease-related, and anthropometric variables were also investigated. The mean age of patients was 61.87 (SD 11.31), while 69.2% were male. The mean glomerular filtration rate was 28.17 (SD 14.03) mL/min/1.73 min^2^, and 70.8% were anemic. Bone/joint pain was the most experienced symptom while the median number of symptoms reported by patients was 5 (IQR 3–7). The mean symptom burden, physical component summary, mental component summary, and kidney-disease-specific component scores were 12.71 (SD 10.45), 68.63 (SD 19.58), 78.53 (SD 18.78), and 81.57 (SD 5.86), respectively. Age was found to be a significant predictor of HRQOL, while hemoglobin level and being a farmer were significant predictors of symptom burden. Our data indicate that CKDu patients in all stages experience at least one symptom affecting all aspects of HRQOL.

## 1. Introduction

Chronic kidney disease (CKD) of uncertain etiology (CKDu) is a form of CKD that emerged during the last two decades, which is not attributed to any conventional risk factors or known etiologies such as hypertension, diabetes, or glomerulonephritis [1,2]. CKDu was described among low-socioeconomic agricultural communities in South Asia (Sri Lanka, and India) and Central America (Nicaragua, El Salvador, Costa Rica, and Guatemala) [3,4,5]. CKDu is clustered in certain geographical locations of Sri Lanka, notably in the dry zone including North Central (NC), Uva, Eastern and North Western provinces, which are the major agricultural regions of the country [1,6]. Epidemiological findings have estimated the point prevalence of CKDu to be between 15.1–22.9% in endemic areas [7], with 70% of CKD cases reported in NC provinces recognized to be CKDu [6]. The disease is predominantly confined to male paddy farmers aged between 40–69 years with low incomes [1,8,9]. The etiology of CKDu is believed to be multi-factorial, and more than 30 scientific hypotheses have been put forward so far to explain the etiological and risk factors of the disease, including heavy metal exposure [7,10], agrochemical toxicity [11], hardness and fluoride content of groundwater [12,13,14], infectious diseases [15], fungal and bacterial toxins [16], dehydration and heat stress [17], and genetic predisposition [18]. Histopathological findings on the disease include interstitial fibrosis, tubular atrophy, and interstitial mononuclear cell infiltration [19,20]. In the past, clinical presentation of the disease was late, and by the time the patients seek treatment, the kidneys have reached end-stage renal disease (ESRD) [21]. Recently initiated community screening programs play an important role in the early identification of the condition. It is difficult to clinically distinguish CKDu from CKD due to other causes [1]. The major focus of researchers has been on the etiology and the screening methods. Nevertheless, there is a scarcity of studies on the care and well-being of the people who are already affected by CKDu. 

Symptom burden has been defined as a subjective, quantifiable prevalence, frequency, and severity of symptoms placing a physiological burden on patients and resulting in multiple negative physical and emotional responses [22,23]. As symptom burden focuses on multiple concurrent symptoms and multidimensional attributes of symptoms, it provides a clear picture of the patient’s experience of the disease [24]. The majority of the studies so far have focused on cancer patients [25], and, recently, attention has been paid to other chronic diseases as well [26,27]. CKD patients experience a multitude of physical and emotional symptoms, such as fatigue, pain, pruritis, shortness of breath, irritability, anxiety, nausea, anorexia, muscle cramps, sexual difficulty, sleep disturbances, and depression due to both the disease and its treatment. These symptoms occur in clusters rather than in isolation [28]. There is growing evidence that reports a high symptom burden among CKD patients at all stages, exerting a negative impact on their quality of life (QOL) [28,29]. Health-related quality of life (HRQOL) is an important aspect which demonstrates the patients’ perceptions of their functioning in different domains including physical, psychological, and social well-being [30]. Compared to in the general population, HRQOL is found to be lower among CKD patients [31], leading to adverse health outcomes [32]. Assessments of symptom burden and HRQOL have been recognized as useful predictors of clinical condition and disease progression of pre-dialysis patients, and their use has been recommended for clinical decision making [28,32,33]. 

Living with a chronic disease such as CKDu requires a variety of adaptations and changes to daily lifestyle and habits, which in turn challenge both the psychological and social wellbeing of patients. As the disease has disproportionately affected rural, poor, male farming communities, financial constrains due to disability, work loss, and out-of-pocket expenditure have had a big impact on both their socio-economic status and psychological wellbeing [34]. Senanayake et al. (2017) assessed the symptom burden and self-perceived severity of symptoms among CKD patients living in Anuradhapura district, Sri Lanka, and found that patients in all stages of CKD experience high symptom burden [35]. Another study which assessed the QOL and symptom burden of CKD patients receiving hemodialysis in Sri Lanka found low QOL, and more than half of the study population reported feelings of worry and sadness [36]. Unfortunately, less is known about the prevalence, severity, and impact of symptoms on the HRQOL of CKDu patients. Therefore, we believe that an understanding of symptom burden, HRQOL, and potential contributing factors in patients with CKDu would be helpful to plan and provide appropriate and timely patient-centered care and to improve the HRQOL of this population. A recent study in Sri Lanka identified level of education, employment status, stage of CKD, dialysis treatment, and presence of co-morbidities as significant predictors of symptom burden [35], and low income and physical and psychological burden as independent predictors of low HRQOL [37] in a CKD/CKDu population. Age, gender, economic status, comorbid medical conditions, stage and duration of kidney disease, body mass index (BMI), and physical activity (PA) were all recorded as possible determinants of HRQOL of CKD patients [38,39,40,41]. On account of the various negative impacts of CKDu on the physical, psychological, and social wellbeing of patients, the disease can be a cause of devastation not only in patients but also their families and relevant agricultural societies. Therefore, this study aimed to assess the prevalence, severity, and burden of symptoms, and HRQOL of CKDu patients and their correlation with socio-demographic, disease-related, anthropometric, and PA characteristics.

## 2. Materials and Methods 

### 2.1. Study Design and Patient Recruitment

A descriptive cross-sectional study was conducted using a randomly selected sample of 120 CKDu patients who attended the renal clinic in Base Hospital, Girandurukotte, Sri Lanka. The clinic register was used as the sampling frame. A survey was carried out during the period from February to May, 2019. The study population consisted of patients who were diagnosed with CKDu by renal biopsy and/or criteria set by the Ministry of Health, Sri Lanka (no past history of diabetes mellitus, chronic or severe hypertension, snake bite, glomerulonephritis, or urological diseases; normal HBA1C (<6.5%), blood pressure <160/100 mmHg untreated or <140/90 mmHg on up to two antihypertensive medications) by a consultant nephrologist (NN). Patients with psychiatric/cognitive disorders or language barriers and patients who refused to participate in the study were excluded from data collection. Ethical clearance was obtained from the ethical review committees of the School of Health Sciences, Niigata University, Japan and the Faculty of Allied Health Sciences, University of Peradeniya, Sri Lanka (AHS/ERC/2018/021). Permission to conduct the study was obtained from relevant authorities in the hospital, clinic and provincial government. Patients were enrolled while they were waiting for their appointments at the renal clinic and, initially, their eligibility to participate in the study was assessed. An information sheet was provided to those who were eligible, prior to the invitation to participate in the survey, and written consent was obtained from all participants. 

### 2.2. Data Collection and Tools

After obtaining patients’ consent to participate in the study, a follow-up home visit was done in order to ensure the accuracy and quality of the data and avoid disturbance of their clinic visit. Basic demographic data was collected and clinical, biochemical, and treatment-related information was extracted from their clinic books, investigation reports, and diagnostic cards if available. The latest available glomerular filtration rate (GFR) within 3 months was used to determine the stage of the disease. According to the Kidney Disease: Improving Global Outcomes paper (KD-IGO), GFR ≥90, 60–89, 30–59, 15–29 and <15 mL/min/1.73 m^2^ were defined as stage I, II, III, IV, and V, respectively [42]. The most recent hemoglobin (Hb) level was recorded referring to patients’ investigation reports within 3 months. In accordance with WHO guidelines, anemia was defined as Hb < 12.0 g/dL in women and Hb < 13.0 g/dL in men [43]. Prevalence, severity and burden of symptoms were assessed using the locally developed and validated CKD Symptom Index—Sri Lanka (CKDSI) [44]. The instrument assessed the prevalence during the 7 days prior to the time of inquiry. Severity of the symptoms was assessed using the following response options; ‘very mild’, ‘mild’, ‘moderate’, ‘severe’, and ‘very severe’, scored from 1–5. A score of zero was allocated for the symptoms not experienced by the patient during the abovementioned time frame, and, summing up the severity scores for all symptoms, the possible symptom burden score for each patient ranged from 0 to 125. We consulted Dr. S. Senanayake; Epidemilogy Unit, Ministry of Health, Colombo, Sri Lanka, the original developer of the CKDSI questionnaire, and obtained permission to use it. 

HRQOL was assessed using the Kidney Disease Quality of Life—Short Form (KDQOL-SF^TM^) version 1.3 questionnaire, developed by RAND cooperation (CA, USA) [45], which has been culturally adapted, modified, and translated into the Sinhala language by Senanayake et al. [46]. This version of the questionnaire has been confirmed as a valid and reliable instrument to assess QOL of CKD patients in Sri Lanka. The instrument basically consists of two components: a kidney-disease-specific component and SF-36. Forty-three items in the kidney-disease-specific component assess 11 domains, including symptoms/problems (12 items), effects of kidney disease (8 items), burden of kidney disease (4 items), work status (2 items), cognitive function (3 items), quality of social interaction (3 items), sexual function (2 items), sleep (4 items), social support (2 items), dialysis staff encouragement (2 items), and patient satisfaction (1 item). SF-36 measures four domains in physical health including physical function (10 items), role limitations caused by physical problems (4 items), pain (2 items), and general health perceptions (5 items), and four domains in mental health including role limitations caused by emotional problems (3 items), social function (2 items), emotional well-being (5 items), energy/fatigue (4 items), and overall health rating (1 item). Answer options are different between questions which usually range from 2 to 7, except for the overall health item, which ranges from 0 to 10. Scores of the different domains and subscales were calculated according to the KDQOL-SF^TM^ scoring manual [47]. First, raw pre-coded numeric values for responses in each item were transformed into a 0 to 100 possible range, where higher scores always reflect better quality of life. Subsequently, items in the same domain or subscale were averaged together to give domain/scale scores ranging from 0 to 100, where higher scores indicate better HRQOL. 

The long form of the international physical activity questionnaire (IPAQ) [48] was translated into the native language and pre-tested prior to use in data collection. The questionnaire assesses frequency, intensity (moderate, vigorous, walking, and sitting), and duration of four domains of PA (work-related, domestic and yard, transportation-related and leisure time) over the past 7 days prior to the inquiry. Reported moderate and vigorous PA time variables below 10 min were scored as 0 min, and those above 180 min were truncated at 180 min, as per the IPAQ scoring protocol [49]. Continuous measurements were expressed as metabolic equivalent of task minutes/week and individuals with ‘high’, ‘moderate’ and ‘low’ PA were identified, based on their categorical scores. Anthropometric measurements, hand grip strength (HGS), and blood pressure (BP) measurements were recorded. Height was measured to the nearest 0.1 cm with an upright portable stadiometer (Seca 213; Seca-Deutschland, Hamburg, Germany), and weight and body composition measurements were obtained using a body composition monitor (Model HBF-220; Omron Healthcare Co. Ltd., Kyoto, Japan). A digital BP monitor (Model MP-126, Berlin, Germany) was used to measure BP, and patients with systolic BP (SBP) of ≥140 mmHg and/or diastolic BP (DBP) of ≥90 mmHg were considered as having hypertension [50]. HGS was measured with a ZAZ electronic hand dynamometer (Zakka-town, Kumagaya, Japan) instructing patients to squeeze the handle with maximum possible force for 3 s by both hands [51]. Body mass index (BMI) was calculated as weight in kilograms divided by height in meters squared (kg/m^2^). BMI cut-off values recommended for Asian populations by WHO expert consultation [52] were used to categorize individuals as underweight (<18.5 kg/m^2^), normal (18.5–22.9 kg/m^2^), overweight (23–27.5 kg/m^2^) and obese (>27.5 kg/m^2^). The skeletal muscle index (SMI) was calculated as body muscle mass divided by the square of body height [53]. 

### 2.3. Statistical Analyses

All statistical analyses were performed using SPSS 23.0 (SPSS, Inc., Chicago, IL, USA). Descriptives were presented as frequencies (*n*) and proportions (%) for categorical variables, mean values and standard deviations (SD), and/or medians and interquartile ranges (IQR) for continuous variables. Significant differences in proportions between groups were calculated by the chi-square test. For the continuous variables, the *t*-test for independent samples or the Mann–Whitney U test was used to compare two groups, depending on the normal/skewed distribution of a particular data set. Differences were regarded as significant at two tailed *p*-value *<* 0.05. Three summary scores in KDQOL^TM^-36—physical component summary (PCS), mental component summary (MCS), and kidney-disease-specific component (KDSC) score—were calculated. Simple and multiple linear regression analyses were then performed to identify the independent predictors for the KDQOL^TM^-36 summary scores and symptom burden score among patients with CKDu. The variables analyzed were age, gender, education level, having a significant other, number of family members, income level, occupation, presence of co-morbidities, family history of CKDu, PA level, CKDu stage, years since diagnosis of CKDu, BMI, fat%, muscle%, HGS, SBP, DBP, GFR, and Hb level. Statistically significant independent variables identified in the simple linear regression were included in the stepwise multiple regression model for determinants of HRQOL and symptom burden. Preliminary analyses were performed to ensure there was no violation of the assumptions of normality, linearity and multicollinearity.

## 3. Results

### 3.1. Patient Characteristics

Out of the 120 participants, 83 (69.2%) were males. The mean (SD) age of the study population was 61.87 (11.31) and the majority were above 50 years of age (*n* = 104, 86.7%). None of the participants had tertiary education and the majority had primary (76.7%) or no education (20.8%). The majority (77.5%) were living with three or more family members and 92.5% were married. Ninety-four percent (93.3%) were engaged in farming, 3.3% were self-employed and the rest were not working. Only seven (5.9%) study participants had more than a 20,000 SLR monthly income, and it was less than 10000 SLR for 35%. Co-morbidities were present among 84 (70%) of the study population. Among the study participants 45 (37.5%), 49 (40.8%), and 24 (20%) were in stage III, IV, and V of CKDu, respectively. Only 25 (20.8%) had family members with CKDu. Only 28.3% were overweight or obese, while 19.2% were underweight. PA level was low among nearly half of the study sample (53.3%). (Table A1). Table 1 lists mean (SD) values of anthropometric and health-related characteristics of the study population. There were significant differences in weight, height, fat%, muscle%, SMI, HGS, and Hb between men and women. Mean GFR was less than 30 mL/min/1.73 min^2^. Mean SBP and DBP were higher than 140 and 90 mm/Hg, respectively, and 2/3 of the study population were hypertensive based on these cut-off values. Mean Hb values for men and women were below the cut-off for both sexes (13 g/dL for men, and 12 g/dL for women), and, based on these values, 85 (70.8%) were anemic. As proportions, 65.1% of men and 83.8% of women were anemic. 

### 3.2. Prevelance, Intrusiveness and Burden of Symptoms

The median number of symptoms reported by patients was 5 (IQR; 3–7), and the mean (SD) was 5.59 (3.73). The majority of patients (*n* = 96, 80%) experienced 1–9 symptoms, with only 5% of patients reporting no symptoms at all. Three (2.5%) patients described having 15 or more symptoms. Prevalence of symptoms among the study population during the period of one week by sex and by stages of CKDu are listed in Table 2. The most prevalent symptoms were bone/joint pain (67.5%; 95% CI 58.3–75.8), loss of appetite (50.8%; 95% CI 41.6–60.1), and lack of energy (47.5%; 95% CI 38.3–56.8). The least prevalent symptoms were changes in skin color and feeling sad (2.5%, 95% CI 0.5–7.1), weight loss (5.8%, 95% CI 2.4–11.6), and hiccups and impotence (6.7%, 95% CI 2.9–12.7). Statistically significant differences between prevalence of symptoms among men and women were found for nausea (M—15.7%, F—32.4%, *p* = 0.037), vomiting (M—2.4%, F—24.3%, *p* < 0.001), and difficulty sleeping (M—42.2%, F—21.6%, *p* = 0.030). There were no significant differences between prevalence of symptoms among CKDu stages. 

In general, perceived severity was ‘mild’ or ‘moderate’ for many symptoms (Table 3). Feeling irritable, difficulty in keeping legs still, muscle cramps, and bone/joint pain were perceived as ‘moderate’ by 16 (38.10%), 17 (37.78%), 16 (34.78%), and 28 (34.75%) of those who experienced the symptoms, while 10 out of 43 patients who experienced difficulty sleeping rated it as ‘severe’. Mean (SD), median (IQR) and range of symptom burden scores were 12.71 (10.45), 10.00 (6.00–17.75), and 0-62, respectively.

### 3.3. Quality of Life 

The domain scores of the KDQOL^TM^-36 were calculated based on individual item responses. Mean scores of patient satisfaction (43.19 ± 10.48), general health perceptions (53.42 ± 18.88), work status (52.50 ± 10.94), and overall health (52.92 ± 14.46) were comparatively lower. Three summary scores, KDSC, PCS, and MCS, were calculated, and mean, median and dispersion scores are summarized in Table 4. Mean PCS was lower compared to mean scores for MCS and KDSC. 

### 3.4. Independent Variables and Their Association with Quality of Life Summary Scores

In simple linear regression, PCS scores were associated with age (*p* < 0.001), having no education (*p* = 0.001), early stages of CKDu (*p* = 0.049), duration of years being diagnosed (*p* = 0.024), GFR (*p* = 0.021), Hb (*p* = 0.001), symptom burden score (*p* < 0.001), HGSL (*p* < 0.001), and HGSR (*p* = 0.003). Simple linear regression of the MCS scores showed age (*p* < 0.001), duration of years being diagnosed (*p* = 0.027), GFR (*p* = 0.020), Hb (*p* = 0.012), symptom burden score (*p* < 0.001), BMI (*p* = 0.010), fat% (*p* = 0.023), and HGSL (*p* = 0.007) as significant determinants. KDSC scores were associated with age (*p* < 0.001), having no education (*p* < 0.001), duration of years being diagnosed (*p* = 0.050), symptom burden score (*p* < 0.001), and HGSL (*p* = 0.041) in simple linear regression (Table 5). Symptom burden scores were associated with engaging in farming (B = −11.830, β = −0.284, *p* = 0.002) and Hb (B = −1.372, β = −0.219, *p* = 0.016) in simple linear regression.

Multiple linear regression was performed to explore how socio-demographic, health-related, and anthropometric characteristics influence HRQOL and symptom burden scores. Variables with *p* values less than 0.05 in simple linear regression, plus gender, were selected. Table 6 summarizes the final significant models obtained by stepwise linear regression. Age was identified as a significant independent predictor, negatively influencing all PCS (*p* < 0.001), MCS (*p* = 0.009), and KDSC (*p* = 0.018) scores. Higher Hb was a significant predictor (*p* < 0.05) of higher scores of PCS (β = 0.177) and lower scores of smptom burden (β = −0.177). Symptom burden score independently influenced all HRQOL scores (PCS; β = −0.417, MCS; β = −0.464, KDSC; β = −0.494). 

## 4. Discussion

This study examined the quality of life and symptom burden of CKDu patients attending a renal clinic in Sri Lanka. To best of our knowledge, this is the first ever study to assess QOL and symptom burden which focused entirely on CKDu patients. As CKDu is a major public health problem in Sri Lanka, these findings are important to gain further understanding of the disease to direct patient management interventions effectively. There was a preponderance of male patients over females, as shown in other studies [9,54,55]. The mean age of the sample was 62, which is higher than the mean ages observed in earlier epidemiological studies related to CKDu [20,54,55]. Muscle mass, HGS, and SMI of both men and women were lower than the cut-off values set by the Asian working group for sarcopenia (AWGS) [56], indicating the prevalence of sarcopenia in this population. However, we did not assess the gait speed of patients to come to a clear conclusion in this regard, and therefore, future research is highly recommended. The mean BMI value of the CKDu subjects was closer to the lower limit of normal BMI levels for Asians, which is consistent with previous findings [57]. Another study found a mean BMI of 22 kg/m^2^ among CKDu patients [58]. A recent study revealed that severity of CKDu is inversely associated with BMI and suggested that low BMI might increase susceptibility to the disease [59]. In the present study, we observed a higher prevalence of anemia among CKDu patients (70.8%), similar to previous findings (72.3%) [60].

One of the important findings of our study is the marked symptom prevalence in CKDu patients, where the vast majority of patients (95%) reported experiencing at least one symptom and 55.8% of them reported having five or more symptoms. Individual analysis of the symptoms revealed that bone/joint pain is the most commonly reported symptom, followed by loss of appetite, lack of energy, muscle cramps, and difficulty in keeping legs still. These findings are compatible with two previous studies conducted in Sri Lanka, which assessed the symptom burden of CKD patients whose etiology may or may not be known. The most commonly reported symptoms were bone/joint pain (87.6%), feeling irritable (78.6%), muscle cramps (77.5%), and lack of energy in one study [35], and tiredness and lack of energy (73.3%), shortness of breath (65.9%), swelling in legs (56.2%), muscle cramps (53.1%) and bone/joint pain (48,9%) in the other study [36]. Both of these studies included patients undergoing dialysis, while there were no dialysis patients included in our study. However, our findings are consistent with other studies which have used CKD patients managed without renal replacement therapy [29,61].

In the present study, the prevalence of symptoms was similar, irrespective of the gender and stage of CKDu. The burden of symptoms in CKD patients at all stages and the importance of symptom assessment even in the early stages of the disease have been pointed out by other studies [29,35]. Brown et al. [29] have reported that men are less likely to report symptoms compared to women and when they do report them usually describe them as less intrusive. Symptoms were frequently reported as mild or moderate in the current study, and the symptom perceived as most severe was difficulty in sleeping. In contrast, a recent study in Sri Lanka reported loss of or decreased libido as the most severe symptom among CKD patients [35]. The symptom burden score of the current study is much lower than the previously reported score by Senanayake et al. (Mean—35.8) [35]. As 77% of the sample in Senanayake et al. [35] were in the advanced stages of the disease and 3.4% were undergoing hemodialysis, the difference in the symptom burden scores might be largely attributed to the differences in patients’ perceptions of the severity of symptoms. Although the symptom burden score was considerably lower in the current study, the fact that 95% of patients are experiencing at least one symptom indicates the importance of incorporating symptom assessment into clinical management from early stages of CKDu.

A few studies have evaluated the QOL of non-dialysis patients. Here, we used previous studies that used the same questionnaire to assess QOL to compare with our findings. The mean KDSC was the highest among QOL quality scores, followed by MCS and PCS, respectively. This points to the fact that, despite the worsening of physical health status, mental health status and kidney-disease-specific QOL aspects are relatively preserved. This may be associated with the chronic nature of the disease, where patients get adapted to the disease, its treatments, and also psychologically to their condition [62,63]. The mean PCS and MCS in our study population was significantly higher compared to Tannor et al. (PCS—43.3; MCS—37.3) [39], Kefale et al. (PCS—38.1; MCS—46) [63] and De Goeij et al. (PCS—54.4; MCS—67.8) [33]. The median summary scores for CKD/CKDu patients reported in a recent study in Sri Lanka were much lower than in the current study (PCS—35.0; MCS—58.4; KDSC—58.4) [37]. This may be attributed to the lower GFR in these study populations compared to the current study. Another study observed PCS of 44.4 and 43.1, and MCS of 46.0 and 47.7 among stage 3 and stage 4 and 5 patients, respectively [62]. Symptom burden score was negatively correlated with all QOL summary scores, suggesting the importance of symptom monitoring in CKDu patients. Symptom burden score was not associated with educational status, CKD stage, or presence of co-morbidities, in contrast to Senanayake et al. [35], who reported those factors as independent predictors of symptom burden among CKD patients in Sri Lanka. Hb and being engaged in farming were the only predictor variables of symptom burden score in the present study. However, when the R^2^ value is considered, these variables explain only 11.2% of the variation in symptom burden score, suggesting that it is influenced by other unrecognized factors.

Among demographic and anthropometric factors, having education, BMI, fat%, and HGS were found to be positively associated with one or more summary scores, while higher age was associated with low QOL. In multiple regression analysis, age was found to be strongly associated with all the summary scores. This finding is in accordance with studies conducted in Brazil [62] and Portugal [64] and different from studies conducted in Ethiopia [63] and Ghana [39]. Similar to previous studies, we found Hb to be a significant predictor of PCS [39,62,63,64] but not of MCS. A prospective study reported a dramatic improvement in QOL of non-dialysis CKD patients with increased Hb levels from <11 to 11–12 g/dl [65]. Having at least primary education was a predictor of a high KDSC score. While relevant literature on KDSC score among non-dialysis patients is limited, this finding may probably be associated with the fact that having at least primary education enhances patients’ ability to comprehend the information given by care givers, thus complying with treatments. Senanayake et al. [37] reported higher education level, being employed, higher income, CKD stage, depression and psychological distress as significant independent predictors of HRQOL among CKD patients in Sri Lanka. HRQOL in the current study, however, was not associated with gender, employment, family income, marital status, nutritional status, or CKD stage. Tannor et al. [39] and Kefale et al. [63] found that both high family income and Hb level were significantly related to better QOL. Similarly, educational status and absence of CKD complications have also been predictors of PCS and MCS, respectively [63]. A study by Cruz et al. [62] found that many socio-demographic factors, including age, ethnicity, gender, professional activity, education, and income, were associated with QOL in relation to clinical factors (co-morbidities, Hb, serum phosphorus level). 

One of the limitations of the study is the relatively non-equal representation between the stages of CKDu to detect significant differences. As patients are usually at late stages of the disease when they are diagnosed, we encountered difficulties in recruiting the subjects in the initial stages of the disease. Secondly, the study was conducted at a single clinic, limiting the extrapolation of results. Finally, the quantitative nature of the data may not highlight patients’ perceptions; thus, studies that consider qualitative assessments, such as interviews and focus group discussions, would have provided a clear understanding of the influence of CKDu on HRQOL.

## 5. Conclusions

In our study population, 95% of patients were suffering from at least one symptom, and high symptom burden was strongly associated with HRQOL. Our findings suggest that patients in early stages of the disease and patients who are not receiving renal replacement therapy suffer from a multitude of symptoms, and, therefore, focus should be given to addressing symptom burden from an earlier stage of the disease. CKDu patients with a higher age were found to have worse QOL. Hb and GFR were associated with PCS and MCS, respectively. Among anthropometric factors, only body fat% was a significant predictor of MCS. Although effects of age cannot be controlled, caregivers should focus on reducing the effects of factors that can be moderated, such as Hb level and symptom burden. In general, assessment of nutritional status, symptom burden and HRQOL of CKDu patients during their routine clinic visits is highly recommended. More longitudinal studies and qualitative studies on these aspects are encouraged. 

## Figures and Tables

**Table 1:** Anthropometric and disease-related characteristics by sex.

Variable	Total (*N* = 120)	Men (*N* = 83)	Women (*N* = 37)	*p*
Weight (kg) ^a^	53.12 (10.02)	54.34 (9.99)	50.40 (9.68)	0.046 *
Height (cm) ^a^	157.83 (7.89)	160.95 (6.51)	150.81 (6.00)	<0.001 *
BMI (kg/m^2^) ^a^	21.25 (3.27)	20.90 (3.21)	22.04 (3.31)	0.077
Fat% ^b^	26.59 (8.41)	23.03 (6.72)	34.58 (5.98)	<0.001 *
Muscle% ^b^	28.05 (4.73)	29.64 (4.73)	24.49 (2.03)	<0.001 *
SMI (kg/m^2^) ^a^	5.90 (1.06)	6.15 (1.14)	5.35 (0.54)	<0.001 *
HGSL ^b^	21.33 (7.54)	23.24 (7.81)	17.05 (4.71)	<0.001 *
HGSR ^a^	21.74 (7.31)	23.59 (7.51)	17.58 (4.74)	<0.001 *
SBP ^a^	144.51 (21.50)	144.69 (22.04)	144.11 (20.51)	0.892
DBP ^a^	95.11 (14.29)	94.82 (14.97)	95.76 (12.81)	0.741
Duration of years since being diagnosed with CKDu ^b#^	6.30 (4.88)	6.36 (4.86)	6.17 (4.99)	0.855
GFR (mL/min/1.73m^2^) ^a^	28.17 (14.03)	29.01 (15.20)	26.96 (10.92)	0.269
Hb (g/dL) ^b^	11.95 (1.67)	12.33 (1.63)	11.09 (1.43)	<0.001 *

a—Independent sample t-test, b—Mann–Whitney U test; Abbreviations: BMI—body mass index; SMI—skeletal muscle index; HGSL—hand grip strength left hand; HGSR—hand grip strength right hand; SBP—systolic blood pressure; DBP—diastolic blood pressure; CKDu—chronic kidney disease of uncertain etiology; GFR—glomerular filtration rate; Hb—hemoglobin; * Significant differences between sexes at *p* < 0.05. ^#^ Data was available for only 107 subjects.

**Table 2:** Prevalence of symptoms among the study population and prevalence by sex and stage in the disease.

Symptom	Frequency (*N* = 120)	Percentage (95% CI)	Prevelance by Sex *n*(%)		Prevelance by Stages of CKDu *n*(%)		
			Male (*N* = 83)	Female (*N* = 37)	Early Stages (*N* = 47)	IV (*N* = 49)	V (*N* = 24)
Loss of appetite	61	50.8 (41.6–60.1)	42 (50.6)	19 (51.4)	20 (42.6)	26 (53.1)	15 (62.5)
Nausea	25	20.8 (14–29.2)	13 (15.7)	12 (32.4)	9 (19.1)	9 (18.4)	7 (29.2)
Vomiting	11	9.2 (4.7–15.8)	2 (2.4)	9 (24.3)	1 (2.1)	7 (14.3)	3 (12.5)
Diarrahea	26	21.7 (14.7–30.1)	20 (24.1)	6 (16.2)	9 (19.1)	9 (18.4)	8 (33.3)
Lethargy	44	36.7 (28.1–45.9)	30 (36.1)	14 (37.8)	16 (34.0)	17 (34.7)	11 (45.8)
Changes in skin colour	3	2.5 (0.5–7.1)	1 (1.2)	2 (5.4)	1 (2.1)	0	2 (8.3)
Swelling of arms and legs	22	18.3 (11.9–26.4)	14 (16.9)	8 (21.6)	6 (12.8)	12 (24.5)	4 (16.7)
Difficulty in breathing	24	20 (13.3–28.3)	19 (22.9)	5 (13.5)	11 (23.4)	5 (10.2)	8 (33.3)
Hiccups	8	6.7 (2.9–12.7)	5 (6)	3 (8.1)	2 (4.3)	4 (8.2)	2 (8.3)
Difficulty keeping legs still	45	37.5 (28.8–46.8)	28 (33.7)	17 (45.9)	17 (36.2)	18 (36.7)	10 (41.7)
Numbness/tingling of hands and feet	24	20 (13.3–28.3)	17 (20.5)	7 (18.9)	8 (17.0)	10 (20.4)	6 (25.0)
Lack of energy	57	47.5 (38.3–56.8)	39 (47)	18 (48.6)	24 (51.1)	21 (42.9)	12 (50.0)
Trouble with memory	22	18.3 (11.9–26.4)	15 (18.1)	7 (18.9)	9 (19.1)	7 (14.3)	6 (25.0)
Weight loss	7	5.8 (2.4–11.6)	4 (4.8)	3 (8.1)	2 (4.3)	2 (4.1)	3 (12.5)
Bone/joint pain	81	67.5 (58.3–75.8)	55 (66.3)	26 (70.3)	32 (68.1)	33 (67.3)	16 (66.7)
Muscle cramps	46	38.3 (29.6–47.6)	31 (37.3)	15 (40.5)	18 (38.3)	19 (38.8)	9 (37.5)
Difficulty concentrating	9	7.5 (3.5–13.8)	4 (4.8)	5 (13.5)	4 (8.5)	3 (6.1)	2 (8.3)
Dry skin	12	10 (5.3–16.8)	8 (9.6)	4 (10.8)	5 (10.6)	2 (4.1)	5 (20.8)
Itching	21	17.5 (11.2–25.5)	14 (16.9)	7 (18.9)	7 (14.9)	7 (14.3)	7 (29.2)
Feeling sad	3	2.5 (0.5–7.1)	2 (2.4)	1 (2.7)	2 (4.3)	1 (2.0)	0
Difficulty sleeping	43	35.8 (27.3–45.1)	35 (42.2)	8 (21.6)	15 (31.9)	18 (36.7)	10 (41.7)
Feeling irritable	42	35 (26.5–44.2)	31 (37.3)	11 (29.7)	16 (34.0)	17 (34.7)	9 (37.5)
Loss/ decreased libido	13	10.8 (5.9–17.8)	10 (12)	3 (8.1)	7 (14.9)	4 (8.2)	2 (8.3)
Impotence	8	6.7 (2.9–12.7)	5 (6)	3 (8.1)	4 (8.5)	4 (8.2)	0
Heartburn	14	11.7 (6.5–18.8)	9 (10.8)	5 (13.5)	8 (17.0)	3 (6.1)	3 (12.5)

**Table 3:** Intrusiveness of symptoms perceived by the study population.

Symptom	*n*	Very mild *n*(%)	Mild *n*(%)	Moderate *n*(%)	Severe *n*(%)	Very Severe *n*(%)
Loss of appetite	61	11 (18.03)	34 (55.74)	10 (16.39)	5 (8.20)	1 (1.64)
Nausea	25	12 (48.0)	9 (36.0)	2 (8.0)	1 (4.0)	1 (4.0)
Vomiting	11	3 (27.27)	6 (54.55)	2 (18.18)	0	0
Diarrahea	26	5 (19.23)	18 (69.23)	3 (11.54)	0	0
Lethargy	44	12 (27.27)	16 (36.36)	13 (29.55)	2 (4.55)	1 (2.27)
Changes in skin colour	3	2 (66.67)	0	1 (33.33)	0	0
Swelling of arms and legs	22	5 (22.73)	12 (54.55)	3 (13.64)	2 (9.09)	0
Difficulty in breathing	24	9 (37.50)	12 (50.00)	2 (8.33)	1 (4.17)	0
Hiccups	8	3 (37.50)	2 (25.00)	2 (25.00)	1 (12.50)	0
Difficulty keeping legs still	45	5 (11.11)	21 (46.67)	17 (37.78)	2 (4.44)	0
Numbness/tingling of hands and feet	24	4 (16.67)	11 (45.83)	7 (29.17)	1 (4.17)	1 (4.17)
Lack of energy	57	13 (22.81)	26 (45.61)	16 (28.07)	2 (3.51)	0
Trouble with memory	22	7 (31.82)	11 (50.00)	1 (4.55)	3 (13.64)	0
Weight loss	7	1 (14.29)	4 (57.14)	2 (28.57)	0	0
Bone/joint pain	81	4 (4.94)	41 (50.62)	28 (34.57)	8 (9.88)	0
Muscle cramps	46	5 (10.87)	18 (39.13)	16 (34.78)	7 (15.22)	0
Difficulty concentrating	9	0	4 (44.44)	3 (33.33)	2 (22.22)	0
Dry skin	12	2 (16.67)	5 (41.67)	2 (16.67)	3 (25.00)	0
Itching	21	3 (14.29)	13 (61.90)	4 (19.05)	1 (4.76)	0
Feeling sad	3	0	2 (66.67)	1 (33.33)	0	0
Difficulty sleeping	43	9 (20.93)	14 (32.56)	9 (20.93)	10 (23.26)	1 (2.33)
Feeling irritable	42	3 (7.14)	16 (38.10)	16 (38.10)	7 (16.67)	0
Loss/decreased libido	13	0	10 (76.92)	2 (15.38)	1 (7.69)	0
Impotence	8	2 (25.00)	4 (50.00)	2 (25.00)	0	0
Heartburn	14	1 (7.14)	9 (64.29)	1 (7.14)	3 (21.43)	0

**Table 4:** Mean and dispersion for Kidney Disease Quality of Life-SF domain scores.

Domain	Mean	SD	Median	IQR	Min	Max
**KDSC score**	81.57	5.86	82.37	78.97–84.97	47.69	93.48
Symptoms/problems	90.63	10.63	93.18	88.64–97.73	22.73	100.00
Effects of kidney disease on daily life	95.21	6.64	96.88	93.75–100.00	63.00	100.00
Burden of kidney disease	83.39	14.35	87.50	75.00–93.75	25.00	100.00
Work status	52.50	10.94	50.00	50.00–50.00	50.00	100.00
Cognitive function	93.61	8.66	93.33	86.67–100.00	47.00	100.00
Quality of social interaction	84.94	11.93	86.67	80.00–93.33	53.00	100.00
Sexual function	90.36	16.14	100.00	75.00–100.00	0.00	100.00
Sleep	73.75	17.88	77.50	63.13–86.88	18.00	100.00
Social suppot	96.25	11.11	100.00	100.00–100.00	33.00	100.00
Hospial staff encouragement	96.35	8.64	100.00	100.00–100.00	75.00	100.00
Patient satisfaction	43.19	10.48	50.00	33.33–50.00	33.00	83.00
PCS score	68.63	19.58	75.31	52.81–85.47	16.88	97.50
Physical functioning	79.21	19.71	85.00	70.00–90.00	15.00	100.00
Role limitations due to physical health problems	70.83	43.40	100.00	6.25–100	0.00	100.00
Pain	71.04	22.64	77.50	55.00–90.00	0.00	100.00
General health perceptions	53.42	18.88	60.00	35.00–70.00	10.00	90.00
MCS score	78.53	18.78	85.81	62.70–94.38	33.25	100.00
Emotional well-being	84.67	15.37	88.00	76.00–100.00	28.00	100.00
Role limitations due to emotional health problems	75.28	41.34	100.00	66.67–100.00	0.00	100.00
Social functioning	79.79	20.57	87.50	62.50–100.00	25.00	100.00
Energy/fatigue	74.38	17.68	75.00	65.00–90.00	10.00	100.00
Overall Health	52.92	14.46	50.00	50.00–60.00	0.00	90.00

Abbreviations: SD—standard deviation; IQR—inter-quartile ratio; Min—minimum; Max—maximum, PCS—physical component summary; MCS—mental component summary, KDSC—kidney-disease-specific component.

**Table 5:** Simple linear regression of socio-demographic, health-related, and anthropometric parameters with PCS, MCS, and KDSC scores.

	PCS					MCS					KDSC				
Predictor	USC B	SE	SC Beta	95% CI for B		USC B	SE	SC Beta	95% CI for B		USC B	SE	SC Beta	95% CI for B	
				Lower bound	Upper bound				Lower Bound	Upper Bound				Lower Bound	Upper Bound
Age	−0.872 **	0.138	−0.503	−1.144	−0.599	−0.601 **	0.143	−0.362	−0.883	−0.319	−0.181 **	0.045	−0.350	−0.270	−0.093
Male gender	−0.376	3.886	−0.009	−8.072	7.320	−6.241	3.683	−0.154	−13.534	1.052	1.412	1.156	0.112	−0.877	3.700
Having no education	−14.053 **	4.226	−0.293	−22.421	−5.685	−4.349	4.220	−0.094	−12.705	4.007	−5.446 **	1.224	−0.379	−7.869	−3.022
Having a significant other	5.871	6.793	0.079	−7.580	19.322	−5.751	6.514	−0.081	−18.650	7.148	1.670	2.033	0.075	−2.356	5.697
Having <5 family members	0.417	3.743	0.010	−6.995	7.828	1.390	3.587	0.036	−5.714	8.494	1.687	1.109	0.139	−0.510	3.883
Monthly income <10000SLR	−2.418	3.756	−0.059	−9.856	5.021	−1.944	3.604	−0.050	−9.082	5.193	−0.083	1.126	−0.007	−2.313	2.147
Engage in farming	3.298	7.189	0.042	−10.937	17.533	4.868	6.886	0.065	−8.768	18.504	1.742	2.147	0.074	−2.510	5.994
Presence of co-morbidities	3.492	3.903	0.082	−4.237	11.222	6.734	3.705	0.165	−0.602	14.071	0.145	1.172	0.011	−2.175	2.466
Having family history of CKDu	−7.263	4.368	−0.151	−15.914	1.388	−4.452	4.219	−0.097	−12.806	3.902	−1.549	1.315	−0.108	−4.152	1.055
Early stages (II and III) of CKDu	7.179 *	3.617	0.180	0.016	14.341	6.629	3.473	0.173	-0.249	13.507	0.498	1.099	0.042	−1.679	2.675
Duration of years after diagnosis	−0.881 *	0.385	−0.218	−1.644	−0.118	−0.835 *	0.373	−0.214	−1.574	−0.097	−0.234 *	0.118	−0.190	−0.469	0.000
GFR	0.295 *	0.126	0.211	0.046	0.543	0.284 *	0.120	0.212	0.045	0.522	0.016	0.038	0.038	−0.060	0.092
Hb	3.621 **	1.027	0.309	1.588	5.654	2.557 *	1.008	0.227	0.561	4.553	0.530	0.319	0.151	−0.102	1.162
SBP	0.000	0.084	0.000	−0.166	0.166	−0.012	0.080	−0.014	−0.171	0.147	0.009	0.025	0.033	−0.041	0.059
DBP	0.095	0.126	0.069	−0.154	0.344	0.062	0.121	0.047	−0.177	0.302	0.016	0.038	0.040	−0.058	0.091
Low PA	−5.688	3.559	−0.146	−12.736	1.360	−4.074	3.430	−0.109	−10.866	2.718	0.295	1.076	0.025	−1.836	2.427
Symptom burden score	−0.797 **	0.156	−0.425	−1.107	−0.488	−0.723 **	0.151	−0.402	−1.023	−0.423	−0.301 **	0.044	−0.536	−0.387	−0.215
BMI	0.698	0.548	0.116	−0.387	1.782	1.343 *	0.514	0.234	0.325	2.361	0.184	0.164	0.102	−0.141	0.508
Body fat%	0.232	0.213	0.100	−0.190	0.655	0.463 *	0.201	0.207	0.064	0.861	−0.017	0.064	−0.024	−0.144	0.110
Body muscle%	0.102	0.381	0.025	−0.653	0.856	−0.251	0.365	−0.063	−0.973	0.472	0.112	0.114	0.090	−0.113	0.336
HGSL	0.815 **	0.227	0.314	0.366	1.264	0.606 **	0.222	0.244	0.166	1.046	0.145 *	0.070	0.187	0.006	0.284
HGSR	0.717 **	0.238	0.268	0.246	1.187	0.412	0.233	0.160	−0.050	0.875	0.131	0.073	0.163	−0.013	0.275

Abbreviations: USC B—unstandardized regression coefficient; SE—standard errors of the regression coefficients; SC Beta—standardized coefficient; CI—confidence interval; PCS—physical component summary; MCS—mental component summary; KDSC—kidney-disease-specific component; CKDu—chronic kidney disease of unknown etiology; GFR—glomerular filtration rate; Hb—hemoglobin; SBP—systolic blood pressure; DBP—diastolic blood pressure; PA—physical activity; BMI—body mass index; HGSL—hand grip strength left hand; HGSR—hand grip strength right hand; * *p* < 0.05 ** *p* < 0.01

**Table 6:** Stepwise multiple regression analysis of independent variables with PCS, MCS, KDSC, and symptom burden scores.

Variable	Unstandardized Coefficients		Standardized Coefficients	95% CI		*p*
	B	SE	Beta	Lower	Upper	
**PCS Score (Adjusted R^2^ = 0.429)**						
Years of age	−0.754	0.135	−0.417	−1.022	−0.486	<0.001
Symptom burden score	−0.782	0.154	−0.383	−1.087	−0.477	<0.001
Hb	2.065	0.873	0.177	0.333	3.798	0.020
**MCS Score (Adjusted R^2^ = 0.360)**						
Symptom burden score	−0.916	0.156	−0.464	−1.225	−0.608	<0.001
Years of age	−0.372	0.140	−0.212	−0.650	−0.094	0.009
Fat%	0.451	0.172	0.205	0.109	0.793	0.010
GFR	0.240	0.105	0.179	0.031	0.449	0.025
**KDSC Score (Adjusted R^2^ = 0.413)**						
Symptom burden score	−0.307	0.047	−0.494	−0.402	−0.213	<0.001
Having no education	−2.951	1.213	−0.203	−5.358	−0.544	0.017
Years of age	−0.109	0.046	−0.198	−0.200	−0.019	0.018
**Symptom burden score (Adjusted R^2^ = 0.112)**						
Engaged in farming	−11.702	3.603	−0.281	−18.837	−4.566	0.002
Hb	−1.347	0.540	−0.215	−2.417	−0.277	0.014

Abbreviations: USC B—regression coefficient; SE—standard errors of the regression coefficients; Beta—standardized coefficient; CI—confidence interval; R^2^—squared multiple correlation coefficients; PCS—physical component summary; MCS—mental component summary; KDSC—kidney-disease-specific component; Hb—hemoglobin; GFR—glomerular filtration rate.

## Appendix A

**Table A1:** Characteristics of the study population.

Variable	Frequency (*n*)	Percentage (%)
Age		
31–50	16	13.3
51–65	53	44.2
65 or older	51	42.5
Gender		
Male	83	69.2
Female	37	30.8
Education level		
No education	25	20.8
Primary	92	76.7
Secondary	3	2.5
Number of family members		
Only 1	1	0.8
Only 2	26	21.7
3–4	50	41.7
5 or more	43	35.8
Marital status		
Married	111	92.5
Widowed	7	5.8
Unmarried	2	1.7
Occupation		
Farming	112	93.3
Self-employed	4	3.3
Retired/not working		3.3
Monthly income (SLR)		
<5000	7	5.8
5001–10000	35	29.2
10001–20000	71	59.2
20001–30000	5	4.2
30001–50000	1	1.7
Presence of co-morbidities		
Yes	36	30
No	84	70
Duration of years being diagnosed with CKDu *		
≥ 1 year	12	11.2
1–5 years	45	42.1
≥ 5 years	21	19.6
≥10 years	29	27.1
Stage of CKDu		
2	2	1.7
3	45	37.5
4	49	40.8
5	24	20
Family members with CKDu		
Yes	25	20.8
No	95	79.2
BMI category		
<18.5	23	19.2
18.5–23	63	52.5
23–27.5	30	25
>27.5	4	3.3
PA level ^#^		
Low	56	53.3
Moderate	25	23.8
High	24	22.9

Abbreviations: SLR—Sri Lankan rupees; CKDu—chronic kidney disease of unknown etiology; BMI—body mass index. * Data was available only for 107 subjects. ^#^ Data was available only for 105 subjects.
